# Pharmacological and non-pharmacological countermeasures to Space Motion Sickness: a systematic review

**DOI:** 10.3389/fncir.2023.1150233

**Published:** 2023-06-16

**Authors:** Akil Khalid, Pragnya P. Prusty, Iqra Arshad, Hannah E. Gustafson, Isra Jalaly, Keith Nockels, Barry L. Bentley, Rahul Goel, Elisa R. Ferrè

**Affiliations:** ^1^Leicester Medical School, University of Leicester, Leicester, United Kingdom; ^2^Department of Audio-Vestibular Sciences, Institute of Health Sciences, Bhubaneswar, India; ^3^Department of Psychology, Royal Holloway University of London, Egham, United Kingdom; ^4^Department of Health and Human Performance, University of Houston, Houston, TX, United States; ^5^University College London Medical School, London, United Kingdom; ^6^Library and Learning Services, University of Leicester, Leicester, United Kingdom; ^7^Cardiff School of Technologies, Cardiff Metropolitan University, Cardiff, United Kingdom; ^8^San Jose State University-NASA Ames Research Center, Moffett Field, CA, United States; ^9^Department of Psychological Sciences, Birkbeck University of London, London, United Kingdom

**Keywords:** Space Motion Sickness (SMS), pharmacological countermeasures, non-pharmacological countermeasures, human space flight, aerospace medicine

## Abstract

**Introduction:**

Space Motion Sickness (SMS) is a syndrome that affects around 70% of astronauts and includes symptoms of nausea, dizziness, fatigue, vertigo, headaches, vomiting, and cold sweating. Consequences range from discomfort to severe sensorimotor and cognitive incapacitation, which might cause potential problems for mission-critical tasks and astronauts and cosmonauts' well-being. Both pharmacological and non-pharmacological countermeasures have been proposed to mitigate SMS. However, their effectiveness has not been systematically evaluated. Here we present the first systematic review of published peer-reviewed research on the effectiveness of pharmacological and non-pharmacological countermeasures to SMS.

**Methods:**

We performed a double-blind title and abstract screening using the online Rayyan collaboration tool for systematic reviews, followed by a full-text screening. Eventually, only 23 peer-reviewed studies underwent data extraction.

**Results:**

Both pharmacological and non-pharmacological countermeasures can help mitigate SMS symptoms.

**Discussion:**

No definitive recommendation can be given regarding the superiority of any particular countermeasure approach. Importantly, there is considerable heterogeneity in the published research methods, lack of a standardized assessment approach, and small sample sizes. To allow for consistent comparisons between SMS countermeasures in the future, standardized testing protocols for spaceflight and ground-based analogs are needed. We believe that the data should be made openly available, given the uniqueness of the environment in which it is collected.

**Systematic review registration:**

https://www.crd.york.ac.uk/prospero/display_record.php?ID=CRD42021244131.

## 1. Introduction

Deep space exploration to the Moon and Mars are no longer in the distant future. Since the first space missions, however, it has been clear that exposure to weightlessness (i.e., microgravity) leads to dramatic functional and structural changes in human physiology, including alterations in the musculoskeletal, cardiovascular, and neural systems (Buckey, 2006). *Space Motion Sickness* (SMS) is a clinical syndrome that affects around 70% of astronauts within the first 72 h of traveling to and returning from microgravity (Heer and Paloski, 2006). SMS symptoms include dizziness, vertigo, headaches, cold sweating, fatigue, nausea, and vomiting. Consequences range from discomfort to severe sensorimotor and cognitive incapacitation. SMS can therefore cause problems during re-entry and emergency exits from a spacecraft; for this reason, no extra-vehicular activities or spacewalks are allowed during the first few days of space missions (Souvestre and Landrock, 2005). Both pharmacological and non-pharmacological countermeasures have been proposed to mitigate SMS symptoms. However, the effectiveness of these countermeasures is still largely debated. The National Aeronautics and Space Administration's (NASA) Human Research Program has recently identified countermeasures against SMS as a critical unaddressed “knowledge gap” (NASA Human Resources Roadmap, 2022). Here we aim to bridge this gap in the knowledge by performing the first systematic review on the effectiveness of pharmacological and non-pharmacological SMS countermeasures. We have collated evidence from published peer-reviewed studies, critically evaluated the current findings, and highlighted potential further research.

Space is a hostile environment: microgravity, extreme temperatures, ionizing radiation and changes in ambient pressure are just some of the stressors space travelers encounter. That is, spaceflight exposes astronauts and cosmonauts to a number of environmental factors that are likely to cause short and long term consequences on human health. For example, exposure to cosmic radiation has dramatic effects on physiological processes (Gundel et al., 1997), and it is associated with a higher risk of cancer and cardiovascular diseases (Townsend, 2005). The lack of atmospheric pressure and solar light severely impacts the neurophysiology processes involved with circadian cycles (Gundel et al., 1997). Microgravity alters different aspects of bodily physiology, including changes in the central nervous system (CNS). Often, these changes occur during and post-flight in the form of neurovestibular problems (Van Ombergen et al., 2017). On Earth, sophisticated organs in the inner ear—the vestibular otoliths—detect gravitational acceleration. When the head moves with respect to terrestrial gravity, the vestibular otoliths shift with the direction of gravitational acceleration, moving the hair cell receptors and signaling to the brain where the head is with respect to the direction of gravity. Vestibular signals are integrated with sensory inputs from vision, and proprioception, to form an *internal model* of terrestrial gravity (Zago and Lacquaniti, 2005; Jörges and López-Moliner, 2017; Lacquaniti et al., 2017). Critically, the internal model created from integrating multiple sensory sources allows a subject to shape their behavior to adapt successfully to the terrestrial gravity environment.

Neurovestibular changes during spaceflight might account for the onset of SMS symptoms. Egorov and Samarin (1970) have considered the asymmetry in the vestibular otoliths as a potential explanation for SMS. In microgravity the vestibular cues for head tilt become irrelevant and lead to a reinterpretation of physical tilt into a translation sensation by the brain (Russomano et al., 2019). This distortion in vestibular signaling is due to asymmetries between the vestibular utricle and saccule on both sides of the head. Critically, the described physiological asymmetries might be aggravated in microgravity conditions leading the central nervous system to misinterpret the signal transmitted by the otolith organs and potentially lose the usual compensatory dynamics for these asymmetries. This might then induce SMS symptoms.

The fluid shifts induced by microgravity may also contribute to SMS (Parker et al., 1983). In space, bodily fluids (i.e., blood and lymph) are dramatically redistributed to the upper parts of the human body due to the absence of hydrostatic pressure. This fluid shift may affect the balance between endolymphatic and perilymphatic pressures in the vestibular labyrinth, causing SMS symptoms (Parker et al., 1983). According to this model, SMS symptoms such as nausea and vomiting might be triggered by unusual patterns of vestibular activity. Importantly, similar symptoms have been described in patients experiencing changes in vestibular structures, such as labyrinthine hydrops or rising intracranial pressure (Noskov and Grigoriev, 1994). However, an account for SMS solely based on fluids shift does not fully explain the symptoms reported by astronauts and cosmonauts. The most destabilizing effects of SMS last from the first to the fifth day of weightlessness and reoccur within the first 10 days after returning to Earth (Oman et al., 1986). In contrast, the shift in fluids develops immediately after entering into orbit and persists until the end of a mission, suggesting alternative etiologies for SMS (Oman et al., 1986).

So far, the most promising approach explaining SMS focuses on sensory conflict (Kohl, 1983). Similarly to terrestrial motion sickness, vestibular and visual cues for spatial orientation might conflict due to the lack of a gravitational reference. In microgravity, signals from the vestibular system no longer provide direct information about gravitational acceleration, which might affect the processing in the brain areas where sensory integration for orientation takes place between vision, proprioception, and vestibular information (e.g., the thalamus, insular cortex, temporoparietal junction, and somatosensory cortices). Accordingly, exposure to this sensory conflict results in SMS symptoms, vestibular illusions, and spatial disorientation (Weerts et al., 2015).

Individual factors such as age, sex, prior flight experience, and individual susceptibility have been suggested to impact the occurrence and severity of SMS. Few studies have explored these factors in SMS directly. Susceptibility to SMS has been shown to decline with flight experience (Golding et al., 2017). Studies have found that, age negatively correlates with motion sickness susceptibility, and females have a higher susceptibility to motion sickness than males (Dobie et al., 2001; Flanagan et al., 2005; Paillard et al., 2013). Differences in hormonal systems and stress expression may explain individual factors in susceptibility (Kohl, 1983), and hereditary susceptibility (Golding et al., 2005) to motion sickness.

Given its impact on space travelers' health, SMS has received much attention in the past years. While SMS is typically experienced in microgravity environments, ground-based research methods have been used to identify its characteristics and investigate potential countermeasures. These methods include centrifugation, parabolic flights, and rotating environments. Centrifugation creates altered gravities through centrifugal force by circular rotations. Parabolic flights elicit short periods of hypergravity (1.8 g), hypogravity (0.38 g, 0.16 g) and microgravity (0 g) through a series of accelerations and free-fall phases. Rotating environments, such as rotating rooms and chairs, disrupt the visual and vestibular information interpreted by the body and brain. Although these ground simulations cannot perfectly mimic spaceflight conditions, they are effective tools to explore SMS.

*Is it possible to overcome SMS?* Pharmacological methods of reducing SMS have been proposed and widely investigated. Several drugs from different pharmacological classes and with varying doses have been explored, including promethazine, scopolamine, dimenhydrinate, prochlorperazine, meclizine, metoclopramide, phenytoin, and lorazepam, among others. A reduction in SMS has been observed after consumption of promethazine or scopolamine during spaceflight (Graybiel, 1976, 1981; Oman et al., 1986; Davis et al., 1993a,b). Similar effects have been reported in parabolic flights (Norfleet et al., 1992; Golding et al., 2017), rotating environments (Graybiel, 1979; Hordinsky et al., 1982; Kohl et al., 1993; Lackner and Graybiel, 1994; Cowings et al., 2000; Dornhoffer et al., 2004; Weerts et al., 2012) and centrifugation (Weerts et al., 2015). However, side effects triggered by promethazine and scopolamine have also been reported, including fatigue, drowsiness, dry mouth, and problems with sensorimotor coordination. Clearly, these side effects are particularly detrimental in high-pressure environments where attention and coordination are critical for performance and survival. Some progress has been made to counteract the adverse side effects of SMS drugs, mainly drowsiness, with the addition of stimulants such as amphetamine (Graybiel, 1981; Hordinsky et al., 1982; Davis et al., 1993a; Kohl et al., 1993). However, more research is needed to understand the underlying mechanisms and effectiveness of using this combined pharmacological approach for the symptomatic treatment of SMS.

Alongside pharmacological approaches, non-pharmacological methods have been explored to mitigate SMS symptoms. These include the use of tilt-transition devices (Harm and Parker, 1994), autogenetic feedback training exercises (Cowings and Toscano, 2000), Virtual Reality training (Stroud et al., 2005), head or body rotation exercises (Reschke et al., 2006; Cloutier and Watt, 2007) and Galvanic Vestibular Stimulation (Dilda et al., 2014). These studies have predominantly focused on building tolerance against SMS. These and reported a reduction in SMS or motion sickness-associated symptoms (Cowings and Toscano, 2000). Thus, non-pharmacological countermeasures might be effective as a pre-flight training countermeasure, particularly in building resilience against SMS. However, the number of studies exploring non-pharmacological countermeasures is severely limited.

Here we performed a systematic review of the published peer-reviewed research on the effectiveness of pharmacological and non-pharmacological countermeasures of SMS. The non-pharmacological countermeasures considered in our systematic review included devices that exposed participants to the stimulus challenges of microgravity, biofeedback training, stroboscopic vision, torso rotation and Galvanic Vestibular Stimulation (Harm and Parker, 1994; Cowings and Toscano, 2000; Stroud et al., 2005; Reschke et al., 2006; Cloutier and Watt, 2007; Dilda et al., 2014). We initially identified 3,207 potential peer-reviewed studies containing SMS keywords, which was reduced to 23 peer-reviewed studies for inclusion following systematic screening (17 on pharmacological countermeasures, five on non-pharmacological countermeasures, and one study that compared both approaches). Results indicate that both pharmacological and non-pharmacological countermeasures have had some success in mitigating SMS symptoms. However, no definitive recommendation can be given about whether one type of countermeasure is more effective given the vast heterogeneity of research methods, the lack of a standardized research approach in evaluating outcomes, and the small sample size overall. Nonetheless, this review provides the first systematic summary of all of the peer-reviewed studies on SMS countermeasures. This can help clarify the state-of-the-art and inform future research on this critical unaddressed knowledge gap.

## 2. Methods

This systematic review was performed under the Preferred Reporting Items for Systematic Reviews and Meta-Analyses (PRISMA) statement (Page et al., 2021). The Librarian (KN), in collaboration with other team members, devised search keywords. Literature searches using Cinahl (via Ebsco), the Cochrane Library, Medline (via Ovid), Scopus, and Web of Science Core Collection databases were performed for relevant articles on 9th April 2021. A follow up search of PubSpace was conducted via PubMed Central on 17th May 2021. Full search strategies are reported in Supplementary material. References from the searches were exported to the Zotero reference management application Zotero (RRID:SCR_013784) to organize references and identify duplicate records, and then exported to Rayyan (Rayyan QCRI (RRID:SCR_017584), a software designed for the screening part of a systematic review through its features to label and sort papers by inclusion or exclusion criterion. Rayyan was therefore used for double-blind screening. A large number of references (3207) were screened for inclusion. Search restrictions were set for peer-reviewed literature, human studies, and the English language. Each article was evaluated for inclusion by four authors (AK, PPP, HEG, and IJ) independently, and all disagreements were resolved by consensus. Out of 3,207 abstracts, 41 full-length articles were screened out, and 23 articles were included as per the inclusion and exclusion criteria stated below. The PRISMA diagram outlining this process is presented in the Supplementary Figure 1.

The inclusion criteria were determined a priori and included specific search terms to ensure all peer-reviewed studies exploring pharmacological and non-pharmacological countermeasures for SMS were captured. Studies outside of our inclusion criteria were rejected, while studies that met the criteria underwent double-blind evaluation. This process ensured that the paper selection was unbiased and systematic. The inclusion criteria for the studies were stated in the form of the Population, Countermeasure, Comparison, Outcomes (PICO) format. They were as follows: (1) P: Adult humans (18 years of age and over) experiencing SMS in altered gravity (spaceflight and ground-based analogs) (2) I: Pharmacological/non-pharmacological (3) C: None (4) O: Miller and Graybiel scale (1968) and subjective self-ratings, or reduced severity/absence of SMS if the countermeasure was done prophylactically (5) Others: Quantitative/Qualitative countermeasure study. The exclusion criteria included: (1) P: People that have had/currently have a stroke, cerebrovascular accidents (CVAs), and peripheral vestibular disorders such as Benign Paroxysmal Positional Vertigo (BPPV), Vestibular Neuritis, Meniere's Disease, Mal de debarquement syndrome); Animal studies, (2) I: None, (3) C: None, (4) O: Terrestrial motion sickness, (5) Others: Non-English Language papers.

The following information was extracted: Title of Paper; Name of the journal; Author (Surname); Year; Funding source (e.g., NASA/European Space Agency [ESA], etc.); Publication Type (Journal article, conference, abstract); Method (Quantitative, Qualitative, Mixed Methods, Other); Altered Gravity Method (Space/Space Analog/Isolation, Confined and Extreme environments [ICE]/Centrifuge/Other); Number of total participants; Number of participants in the control group; Types of participants (Astronauts, cosmonaut, healthy adults, etc.); Participants (Male: Female ratio if available); Participant's age (mean age, standard deviation/range of age); Number of trained participants/naïve; Was any inclusion/exclusion criteria applied to participants? (Yes/No/Maybe); Group allocation; How were participants divided into groups (if applicable); Was there a control group (Yes/No/Not Sure); Duration of exposure in microgravity/altered gravity environment (making sure time is standardized, i.e., all in hours/days/weeks); Measurement timeline (e.g., pre-flight/in-flight/post-flight); How was SMS measured (self-report, biomarkers etc.); Note on any data exclusion and why; Type of countermeasure used for SMS (pharmacological or non-pharmacological); Name of countermeasure used for SMS (if pharmacological, include mode of administration and dose); Duration of countermeasure used for SMS; How was SMS affected (+ meaning reduced, – meaning increased, or = meaning negligible), Double signs with space between them if statistically significant; If effective, specify which symptoms were improved; Any side effects of countermeasure; Any secondary outcomes and if so, main result(s) of this; Advantages of countermeasure; Any limitations of the countermeasure itself. The type of study was also identified.

## 3. Results

Our systematic review focused on the effectiveness of pharmacological and non-pharmacological countermeasures to counteract SMS. Notably, only one study (Cowings and Toscano, 2000) directly compared both types of countermeasures. Overall, considerable heterogeneity emerged in the characteristics and methodologies of the studies (e.g., sample size, type, and duration of the altered gravity methods and how the effect of SMS was measured). Of the 23 studies, 17 looked at pharmacological countermeasures (Table 1). There were 15 different drugs or drug combinations used, which varied in dosage, timing, and mode of administration. The most studied drugs and their doses were 0.4 mg of oral scopolamine and 25 mg or 50 mg of oral or intramuscular promethazine. Every study reported a beneficial effect on SMS, defined mainly as a reduction in SMS symptoms experienced by participants. In 10 of the 17 studies, this result was reported as statistically significant. Golding et al. (2017) are notable for their study characteristics of a relatively large sample size of 246, including female participants, and choice of parabolic flight over a space analog (Golding et al., 2017). The most common side effect was drowsiness reported with promethazine consumption.

**Table 1:** Summary of study characteristics for pharmacological countermeasures in chronological order.

**Study**	**Sample size**	**M:F ratio**	**Spaceflight/analog**	**Type of study**	**Specific countermeasure**	**Main findings**
Graybiel (1976)	31	N/A	Spaceflight Slow rotation room Parabolic flight	Descriptive	Promethazine 25 mg/ephedrine 25 mg Promethazine 25 mg/ephedrine 50 mg Scopolamine 0.3 mg/ephedrine 25 mg Scopolamine 0.3 mg/d-amphetamine sulfate 5 mg Scopolamine 0.3 mg Scopolamine 0.6 mg Dimenhydrinate 50 mg (Route of administration for all is N/A)	Promethazine 25 mg/ephedrine 25 mg were both beneficial in alleviating SMS symptoms.
Graybiel (1979)	40	N/A	Slow rotation room	Descriptive	Transdermal scopolamine (Dose N/A) Oral scopolamine 0.3 mg Oral promethazine 25 mg/ephedrine 25 mg Oral promethazine 25 mg/ephedrine 12.5 mg Oral prochlorperazine 10 mg/isopropamide 5 mg Oral meclizine 25 mg/ephedrine 25 mg Oral dimenhydrinate 25 mg/ephedrine 25 mg	Oral promethazine 25 mg/ephedrine 25 mg showed the best response.
Graybiel (1981)	6	6:0	Spaceflight	Descriptive	Scopolamine 0.35 mg/dexedrine 50 mg Promethazine 25 mg/ephedrine 50 mg (Route of administration for all is N/A)	Scopolamine had confirmed efficacy when given pre-flight.
Hordinsky et al. (1982)	9	9:0	Rotary chair	Repeated measures Double blind Placebo	Transdermal scopolamine 1.5 mg Oral scopolamine/dextroamphetamine combination 0.4 mg/5 mg Oral promethazine and ephedrine 25 mg/25 mg Intramuscular promethazine, 25 mg	While oral promethazine/ephedrine was the most effective at alleviating SMS, transdermal scopolamine was recommended as it is effective with fewer side effects.
Oman et al. (1986)	4	N/A	Spaceflight	Descriptive Repeated measures	Oral scopolamine 0.4 mg/dexedrine 2.5 mg Promethazine 25 mg/ephedrine 25 mg (Route of administration is N/A) Metoclopramide 10 mg (Route of administration for all is N/A)	Scopolamine and promethazine were effective.
Norfleet et al. (1992)	21	21:0	Parabolic flight	Repeated measures Crossover design Placebo Single blind	Buccal scopolamine 1 mg	Buccal scopolamine significantly lowered scores for nausea and vomiting compared to a placebo.
Kohl et al. (1993)	53	53:0	Rotary chair	Repeated measures Crossover design Double blind Placebo	Oral doxepin 70 mg Oral scopolamine 0.4 mg/amphetamine 5 mg	Both medications showed a significant difference in adaptation to stressful Coriolis stimuli compared to placebo.
Davis et al. (1993a)	96	N/A	Spaceflight	Repeated measures	Intramuscular promethazine 50 mg	Intramuscular promethazine after symptom development was significantly effective in immediate symptom relief.
Davis et al. (1993b)	34	N/A	Spaceflight	Repeated measures Quasi-experimental groups	Scopolamine 0.4 mg/dextroamphetamine 2.5–5.0 mg Promethazine 25–50 mg	Promethazine was most effective.
Lackner and Graybiel (1994)	18	18:0	Rotary room	Experimental groups Repeated measures Placebo	Oral promethazine 50 mg	Oral promethazine delayed the onset of nausea compared to a placebo group.
Knox et al. (1994)	6-13	N/A	Parabolic flight	Repeated measures Double blind Placebo Crossover design	Oral phenytoin 500–1,200 mg pre-flight Oral phenytoin 100–200 mg as needed	Oral phenytoin was effective as only 7.7% of participants had level 3 or greater nausea (i.e., severe, performance of tasks affected or vomiting).
Cowings et al. (2000)	12	12:0	Rotary chair	Repeated measures Double blind Placebo	Intramuscular promethazine 25 mg Intramuscular promethazine 50 mg	Both doses significantly increased motion sickness tolerance compared to the placebo. The incidence of performance-impaired subjects almost doubled with 50 mg dose compared to 25 mg dose.
Dornhoffer et al. (2004)	75	45:27	Rotary chair	Repeated measures Prospective study Double blind	Oral lorazepam 1 mg Oral meclizine 25 mg Oral promethazine 25 mg Oral scopolamine 0.4 mg	Oral scopolamine was significantly more effective.
Weerts et al. (2012)	16	7:9	Rotary chair	Repeated measures Single blind Placebo	Oral lorazepam 1 mg Oral meclizine 25 mg Oral promethazine 25 mg Oral scopolamine 0.4 mg	Oral meclizine, oral scopolamine, and oral lorazepam were recommended for future studies.
Weerts et al. (2014)	20	20:0	Rotary chair	Repeated measures Double blind Placebo	Oral meclizine 25 mg Oral promethazine 25 mg/d-amphetamine 10 mg Oral dimenhydrinate 40 mg/cinnarizine 25 mg	Oral meclizine and oral dimenhydrinate/cinnarizine were recommended for future studies.
Weerts et al. (2015)	19	19:0	Unilateral centrifugation	Repeated measures Double blind Placebo	Intranasal scopolamine 0.4 mg	Intranasal scopolamine significantly reduced vestibular ocular reflex gain and total calorific response during electronystagmographic recording.
Golding et al. (2017)	246	192: 54	Parabolic flight	Experimental groups	Subcutaneous scopolamine 0.175 mg or less	Subcutaneous scopolamine reduced vomiting compared to flyers who did not take medication.

Of the 23 studies, six looked at non-pharmacological countermeasures (Table 2). The specific types were devices that exposed participants to the stimulus challenges of microgravity, biofeedback training, stroboscopic vision, torso rotation and Galvanic Vestibular Stimulation (Harm and Parker, 1994; Cowings and Toscano, 2000; Stroud et al., 2005; Reschke et al., 2006; Cloutier and Watt, 2007; Dilda et al., 2014). Unlike the pharmacological studies, all but the stroboscopic vision study carried out their countermeasure prophylactically rather than therapeutically (Harm and Parker, 1994; Cowings and Toscano, 2000; Stroud et al., 2005; Cloutier and Watt, 2007; Dilda et al., 2014). Like the pharmacological studies, each of the six non-pharmacological studies also reported a beneficial effect of SMS by reducing symptoms. In four of them, this result was reported as statistically significant.

**Table 2:** Summary of study characteristics for non-pharmacological countermeasures in chronological order.

**Study**	**Sample size**	**M:F ratio**	**Spaceflight/analog**	**Type of study**	**Specific countermeasure**	**Main finding**
Harm and Parker (1994)	27	N/A	Tilt-transition device	Repeated measures	Pre-flight adaptation training	Tolerance time increased as motion sickness symptoms decreased over time.
Cowings and Toscano (2000)	33	33:0	Rotary chair	Repeated measures Double blind Placebo	Autogenic-feedback training exercise (AFTE) Intramuscular promethazine 25 mg Intramuscular promethazine 50 mg	AFTE was significantly more effective than either dose of intramuscular promethazine.
Stroud et al. (2005)	30	17:13	Virtual reality DOME system (Device for orientation and motion environment)	Experimental groups Repeated measures	Variable training Non-variable training	Variable training resulted in fewer nausea symptoms.
Reschke et al. (2006)	32	19:13	Head movements	Repeated measures Crossover design Control	Stroboscopic vision and shutter glasses	While flashing, the strobe-illuminated environment and the shutter glasses significantly lowered motion sickness.
Cloutier and Watt (2007)	25	5:20	Head nodding	Repeated measures	Torso rotation	Torso rotation significantly decreased motion sickness.
Dilda et al. (2014)	10	7:3	Galvanic vestibular stimulation	Repeated measures	Galvanic vestibular stimulation	During 12 weekly GVS exposures, body sway and postural dynamics were comparable to pre-GVS baseline at 7–8 weeks into the 12-week programme. The effect was still maintained 6 months post-GVS exposure.

## 4. Discussion

Here we have systematically reviewed the available literature to explore the effectiveness of both pharmacological and non-pharmacological countermeasures against SMS. Despite the potential consequences of SMS on crewmember well-being and space mission success, findings remain inconsistent and contradictory in places. While positive results were found in both types of countermeasures, no clear and reliable evidence emerged about which countermeasure is most effective for addressing SMS symptoms. Evidently, across the pharmacological and non-pharmacological studies, a lack of a standardized protocol makes comparisons within and between the approaches almost impossible.

The pharmacology literature dramatically shows the lack of consistency and standardized approaches. Some studies have administered drugs such as scopolamine through transdermal means (Graybiel, 1979; Hordinsky et al., 1982) and others via oral intake (Oman et al., 1986; Davis et al., 1993a; Dornhoffer et al., 2004). Differences in administration may affect uptake and how quickly the drug impacts SMS symptoms. In addition, varying doses were reported in the literature with ambiguity around the time of administration, the number of doses, and how soon after SMS symptoms were measured. Little effort has been made to discriminate when the administration of drugs is most effective in relation to flight times, e.g., pre-, during, or post-flight. Research has also heavily focused on scopolamine and promethazine with little exploration of alternatives, given their side effects. It is, therefore, difficult to conclude which pharmaceutical drug is most effective in reducing SMS and when.

Similarly, drawing concrete conclusions or comparing non-pharmacological countermeasures is also tricky. A noticeable lack of replication or evaluation puts into question the reliability of these countermeasures in reducing SMS. More research and replication are needed, even using ground-based analogs that are more widely and relatively cheaply accessible, to explore the effectiveness of non-pharmacological countermeasures, to assess whether the resistance against SMS is genuinely achieved and whether a consistent reduction of symptoms is experienced.

SMS can be debilitating, and more efforts are needed to address symptoms. More consideration should be given to adopting a combined approach, not only of pharmaceuticals but also of pharmacological and non-pharmacological countermeasures. For instance, non-pharmacological countermeasures could increase tolerance to SMS during pre-flight training, while pharmacological countermeasures could be applied in-flight and post-flight to ease symptoms directly. Previous research has also primarily focused on addressing nausea-related symptoms of SMS. While this is crucial, SMS encompasses a range of symptoms that can affect the operational effectiveness of astronauts, including increased body warmth, sweating, loss of appetite, fatigue, and anorexia (Heer and Paloski, 2006). More efforts should be made to address other SMS symptoms as well.

Comparisons within each countermeasure domain are also complicated since some studies have explored SMS in actual spaceflight conditions, whereas others have used ground-based analogs. For example, comparing symptoms of SMS during an International Space Station (ISS) mission with a terrestrial rotary chair may not be a valid comparison given the stark differences in physiological, musculoskeletal, neurobiological factors and potentially different causative elements for SMS in both cases. Despite gravity always being present, ground-based analogs and simulations have been widely adopted across space research and could help develop effective countermeasures against SMS. However, standardization is required if fair comparisons are to be made.

Importantly, most of the current literature relies heavily on self-reported measures to capture the prevalence and severity of SMS symptoms (Hordinsky et al., 1982; Davis et al., 1993a; Kohl et al., 1993; Knox et al., 1994; Cowings and Toscano, 2000; Dornhoffer et al., 2004; Stroud et al., 2005; Reschke et al., 2006; Cloutier and Watt, 2007). Although widely used, this qualitative approach may be susceptible to bias impacting the accuracy and validity of the findings. More objective and quantitative methods of measuring SMS symptoms should be considered, such as physiological measures (e.g., heart rate, respiration, or skin conductance). These may act as precursors to actual SMS and could help develop targeted countermeasures while providing a more holistic and representative understanding of the onset and prevalence of SMS. If adequately validated, deviations in physiological measures can also be integrated into some early warning systems.

Our systematic review reveals that the current research suffers from severely restricted and biased samples. Participant samples are male-dominant and consist primarily of highly trained personnel, often recruited multiple times across different studies. As shown in Table 1, several studies consisted of all male participants. Addressing the lack of gender balance is critical since there are key hormonal and physiological differences between males and females that may impact the onset and severity of SMS symptoms (Reschke et al., 2014). With more efforts by space agencies and commercial companies to recruit females for crewed missions (Guzman, 2019), it is crucial to understand how SMS impacts both genders.

The reviewed literature provides a promising indication that both pharmacological and non-pharmacological approaches can be used to address SMS; however, given some of the challenges of doing space research mentioned above, our review paints an incomplete and inconsistent picture of the effectiveness of current countermeasures. Despite our systematic approach, no single countermeasure appears superior in addressing SMS. Importantly, there is an apparent lack of research into non-pharmacological studies, which might undermine its potential. Some consideration should also be given to combining the strengths of pharmacological and non-pharmacological approaches to reduce SMS symptoms maximally. Critically, the findings presented here are a snapshot of the current SMS literature. The vast majority of data generated by leading space agencies may not be published due to clinical confidentiality, time limitations, or non-significant results. To better understand SMS, researchers need to move toward an open science and transparent approach whereby data and peer-reviewed papers are made widely available sooner than later.

SMS's high prevalence and potentially lethal consequences reinforce the need to quickly find effective countermeasures to alleviate symptoms and ensure the success of human space missions. There is no doubt that long duration spaceflight and exposure to microgravity will increase the risk of developing SMS and, therefore symptoms that might very likely impact the physical and cognitive functioning of space travelers. Understanding the mechanisms of SMS and developing countermeasures is imperative for safety, health, and productivity of space crewmembers during future missions to the Moon, Mars, and beyond.

## Data availability statement

The original contributions presented in the study are included in the article/Supplementary material, further inquiries can be directed to the corresponding author.

## Author contributions

AK, PPP, IA, and IJ identified the keywords for scoping searches. KN carried out database searches using a search strategy. AK, PPP, IJ, and HG screened papers. AK, PPP, IA, HG, IJ, and BB completed data extraction on included papers and contributed to manuscript writing. EF and RG provided guidance throughout the systematic review and helped with manuscript editing. All authors read and approved the final manuscript.
