# Supplementary material for: Pharmacological and non-pharmacological countermeasures to Space Motion Sickness: a systematic review
Source: Front Neural Circuits. 2023 Jun 16;17:1150233. doi: 10.3389/fncir.2023.1150233 (PMC10311550; doi:10.3389/fncir.2023.1150233)
Supplement: Supplementary file 1 [file Data_Sheet_1.docx]

**Supplementary Materials**

**Supplementary Figures**

**
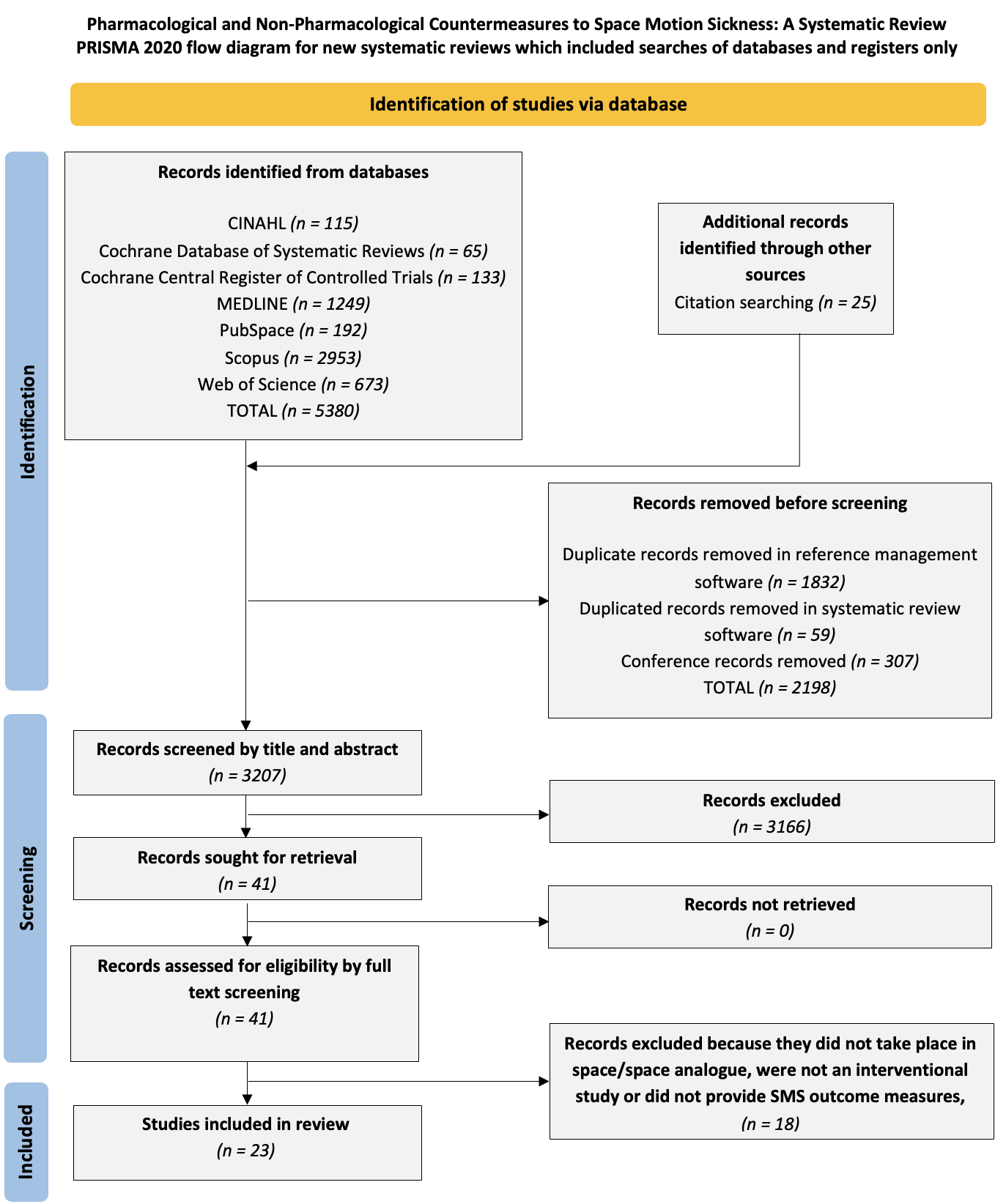
**Figure 1

**Appendix – Search Strategies**

Cinahl

S1           (space N2 sickness)

S2           (MH “Aerospace Medicine”)

S3           (MH “space flight”)

S4           (extraterrestrial or “extra terrestrial”)

S5           (“space flight*” or spaceflight*)

S6           ((space or moon or lunar) N2 (travel* or flight*))

S7           ((space or moon or lunar) N2 explor*)

S8           “space adaptation syndrome”

S9           (astronaut* or cosmonaut*)

S10         (MH “Gravitation+”)

S11         (gravit* or hypergrav* or hypograv* or micrograv* or “hyper grav*” or “hypo grav*” or “micro grav*”)

S12         weightless*

S13         (nausea* or nauseous)

S14         vomit*

S15         (MH “Nausea and Vomiting+”)

S16         sickness

S17         (MH “Motion Sickness”)

S18         (spatial N2 disorientation)

S19         (MH “Perception+”)

S20         ((sensorimotor or vestib*) N2 (disturb* or conflict* or signal*))

S21         ((visual or vestibular or canal or otolith) N2 (disturb* or conflict* or signal*))

S22         sopite

S23         S2 OR S3 OR S4 OR S5 OR S6 OR S7 OR S8 OR S9 OR S10 OR S11 OR S12

S24         S13 OR S14 OR S15 OR S16 OR S17 OR S18 OR S19 OR S20 OR S21 OR S22

S25         S23 AND S24

S26         S1 OR S25

S27         (MH “Miscellaneous Drugs and Agents+”)

S28         (parab* N2 flight*)

S29         (MH “Centrifugation”)

S30         centrifug*

S31         (MH “Head-Down Tilt”) OR (MH “Tilt-Table Test”)

S32         tilt*

S33         “virtual reality”

S34         (MH “Virtual Reality+”)

S35         “carbon dioxide”

S36         (MH “Carbon Dioxide”)

S37         hypoxia

S38         (MH “Anoxia”) OR (MH “Hypoxia, Brain+”)

S39         exercise*

S40         (MH “Exercise+”)

S41         “rotary chair”

S42         (gvs or “galvanic vestibular stimulation”)

S43         (treat* or intervention* or counter*)

S44         (Pharmacokinetic* or Drug or drugs or Pharmacology or Medical or Nasal or Medication)

S45         (Scopolamine or Promethazine or Compazine or Phenergan or Ephedrine or Dextroamphetamine or Phenergan or Dexedrine or Meclizine or Cinnarizine or Dimenhydrinate)

S46         (Antidopaminergic* or “anti dopaminergic*” or Antihistaminergic* or “anti histaminergic*” or Anticholinergic* or “anti cholinergic*” or Placebo or Antivertig* or “anti vertig*”)

S47         S27 OR S28 OR S29 OR S30 OR S31 OR S32 OR S33 OR S34 OR S35 OR S36 OR S37 OR S38 OR S39 OR S40 OR S41 OR S42 OR S43 OR S44 OR S45 OR S46

S48         S26 AND S47

Cochrane

#1    [mh “space motion sickness”]

#2    (space NEAR/2 sickness):ti,ab,kw

#3    [mh “Aerospace Medicine”]

#4    [mh “space flight”]

#5    [mh ^“Extraterrestrial environment”]

#6    (extraterrestrial or “extra terrestrial”):ti,ab,kw

#7    (“space flight*” or spaceflight*):ti,ab,kw

#8    ((space or moon or lunar) NEAR/2 (travel* or flight*)):ti,ab,kw

#9    ((space or moon or lunar) NEAR/2 explor*):ti,ab,kw

#10    “space adaptation syndrome”:ti,ab,kw

#11    [mh Astronauts]

#12    (astronaut* or cosmonaut*):ti,ab,kw

#13    [mh Gravitation]

#14    (gravit* or hypergrav* or hypograv* or micrograv* or "hyper grav*" or "hypo grav*" or "micro grav*"):ti,ab,kw

#15    weightless*:ti,ab,kw

#16    (nausea* or nauseous):ti,ab,kw

#17    vomit*:ti,ab,kw

#18    [mh ^Nausea]

#19    [mh ^vomiting] or [mh “vomiting, anticipatory”]

#20    sickness:ti,ab,kw

#21    [mh ^“Motion Sickness”]

#22    (spatial NEAR/2 disorientation):ti,ab,kw

#23    [mh “space perception”]

#24    ((sensorimotor or vestib*) NEAR/2 (disturb* or conflict* or signal*)):ti,ab,kw

#25    ((visual or vestibular or canal or otolith) NEAR/2 (disturb* or conflict* or signal*)):ti,ab,kw

#26    sopite:ti,ab,kw

#27    #1 OR #2

#28    {OR #3-#15}

#29    {OR #16-#26}

#30    #28 and #29

#31    #27 or #30

#32    [mh "chemicals and drugs"]

#33    (parab* NEAR/2 flight*):ti,ab,kw

#34    [mh Centrifugation] or centrifug*:ti,ab,kw

#35    [mh “Head-Down Tilt”] or “head down bed rest”:ti,ab,kw

#36    tilt*:ti,ab,kw

#37    “virtual reality”:ti,ab,kw OR [mh "virtual reality"]

#38    “carbon dioxide”:ti,ab,kw or [mh “Carbon Dioxide”]

#39    hypoxia:ti,ab,kw or [mh Hypoxia]

#40    [mh ^Exercise] or exercise:ti,ab,kw

#41    “rotary chair”:ti,ab,kw

#42    (gvs or “galvanic vestibular stimulation”):ti,ab,kw

#43    (treat* or intervention* or counter*):ti,ab,kw

#44    (Pharmacokinetic* or Drug or drugs or Pharmacology or Medical or Nasal or Medication):ti,ab,kw

#45    (Scopolamine or Promethazine or Compazine or Phenergan or Ephedrine or Dextroamphetamine or Phenergan or Dexedrine or Meclizine or Cinnarizine or Dimenhydrinate):ti,ab,kw

#46    (Antidopaminergic* or “anti dopaminergic*” or Antihistaminergic* or “anti histaminergic*” or Anticholinergic* or “anti cholinergic*” or Placebo or Antivertig* or “anti vertig*”):ti,ab,kw

#47    {OR #32-#46}

#48    #31 AND #47

Medline

1    exp space motion sickness/

2    (space adj2 sickness).mp.

3    Aerospace Medicine/

4    exp space flight/

5    Extraterrestrial environment/

6    (extraterrestrial or extra terrestrial).mp.

7    (space flight* or spaceflight*).mp.

8    ((space or moon or lunar) adj2 (travel* or flight*)).mp.

9    ((space or moon or lunar) adj2 explor*).mp.

10    space adaptation syndrome.mp.

11    Astronauts/

12    (astronaut* or cosmonaut*).mp.

13    exp Gravitation/

14    (gravit* or hypergrav* or hypograv* or micrograv* or hyper grav* or hypo grav* or micro grav*).mp.

15    weightless*.mp.

16    (nausea* or nauseous).mp.

17    vomit*.mp.

18    Nausea/

19    vomiting/ or vomiting, anticipatory/

20    sickness.mp.

21    Motion Sickness/

22    (spatial adj2 disorientation).mp.

23    exp space perception/

24    ((sensorimotor or vestib*) adj2 (disturb* or conflict* or signal*)).mp.

25    ((visual or vestibular or canal or otolith) adj2 (disturb* or conflict* or signal*)).mp.

26    sopite.mp.

27    1 or 2

28    or/3-15

29    or/16-26

30    28 and 29

31    27 or 30

32    limit 31 to clinical trial, all

33    exp "chemicals and drugs (non mesh)"/

34    (parab* adj2 flight*).mp.

35    exp Centrifugation/ or centrifug*.mp.

36    Head-Down Tilt/ or head down bed rest.mp.

37    tilt*.mp.

38    virtual reality.mp. or Virtual Reality/

39    carbon dioxide.mp. or Carbon Dioxide/

40    hypoxia.mp. or exp Hypoxia/

41    Exercise/ or exercise.mp.

42    rotary chair.mp.

43    (gvs or galvanic vestibular stimulation).mp.

44    (treat* or intervention* or counter*).mp.

45    (Pharmacokinetic* or Drug or drugs or Pharmacology or Medical or Nasal or Medication).mp.

46    (Scopolamine or Promethazine or Compazine or Phenergan or Ephedrine or Dextroamphetamine or Phenergan or Dexedrine or Meclizine or Cinnarizine or Dimenhydrinate).mp.

47    (Antidopaminergic* or anti dopaminergic* or Antihistaminergic* or anti histaminergic* or Anticholinergic* or anti cholinergic* or Placebo or Antivertig* or anti vertig*).mp.

48    or/33-47

49    31 and 48

50    limit 49 to animals

51    49 not 50

PubSpace

(nausea[tw] OR nauseous[tw] OR vomit[tw] OR Nausea[MeSH Terms] OR vomiting[MeSH Terms] OR vomiting, anticipatory[MeSH Terms] OR sickness[tw] OR Motion Sickness[MeSH Terms] OR space motion sickness[MeSH Terms]) AND nasa funded[Filter]

Scopus

(TITLE-ABS-KEY("space motion sickness" OR (space W/2 sickness)) OR TITLE-ABS-KEY(("aerospace medicine" OR "space flight" or "extravehicular activity" or spacecraft or "space craft" OR "Extraterrestrial environment" OR extraterrestrial or "extra terrestrial" OR "space flight*" or spaceflight* OR ((space or moon or lunar) W/2 (travel* or flight*)) OR ((space or moon or lunar) W/2 explor*)) OR "space adaptation syndrome" OR astronaut* or cosmonaut* OR gravit* or hypergrav* or hypograv* or micrograv* or "hyper grav*" or "hypo grav*" or "micro grav*" OR weightless*) AND (nausea* or nauseous OR vomit* OR sickness OR (spatial W/2 disorientation) OR ((space or depth or form or distance or size) W/2 perception) OR "contrast sensitivity" OR "vision disparity" OR ((sensorimotor or vestib*) W/2 (disturb* or conflict* or signal*)) OR ((visual or vestibular or canal or otolith) W/2 (disturb* or conflict* or signal*)) OR sopite)) AND (TITLE-ABS-KEY((parab* W/2 flight*) OR Ultracentrifuge* OR centrifug* OR "head down bed rest" OR tilt* OR "virtual reality" OR "carbon dioxide" OR hypoxia OR exercise OR "rotary chair" OR gvs or "galvanic vestibular stimulation" OR treat* or intervention* or counter* OR Pharmacokinetic* or Drug or drugs or Pharmacology or Medical or Nasal or Medication OR Scopolamine or Promethazine or Compazine or Phenergan or Ephedrine or Dextroamphetamine or Phenergan or Dexedrine or Meclizine or Cinnarizine or Dimenhydrinate OR Antidopaminergic* or "anti dopaminergic*" or Antihistaminergic* or "anti histaminergic*" or Anticholinergic* or "anti cholinergic*" or Placebo or Antivertig* or "anti vertig*"))

Web of Science Core Collection

#1 TOPIC: (“space motion sickness” OR (space NEAR/2 sickness))

#2 TOPIC: ("aerospace medicine" OR "space flight" or "extravehicular activity" or spacecraft or "space craft" OR "Extraterrestrial environment" OR extraterrestrial or "extra terrestrial" OR "space flight*" or spaceflight* OR "space adaptation syndrome" OR astronaut* or cosmonaut* OR gravit* or hypergrav* or hypograv* or micrograv* or "hyper grav*" or "hypo grav*" or "micro grav*" OR weightless*)

#3 TOPIC: (((space or moon or lunar) NEAR/2 (travel* or flight*)) OR ((space or moon or lunar) NEAR/2 explor*))

#4 TOPIC: (nausea* or nauseous OR vomit* OR sickness OR (spatial NEAR/2 disorientation) OR ((space or depth or form or distance or size) NEAR/2 perception) OR “contrast sensitivity” OR “vision disparity” OR ((sensorimotor or vestib*) NEAR/2 (disturb* or conflict* or signal*)) OR ((visual or vestibular or canal or otolith) NEAR/2 (disturb* or conflict* or signal*)) OR sopite)

#5 TOPIC: ((parab* NEAR/2 flight*) OR Ultracentrifuge* OR centrifug* OR "head down bed rest" OR tilt* OR "virtual reality" OR "carbon dioxide" OR hypoxia OR exercise OR "rotary chair" OR gvs or "galvanic vestibular stimulation" OR treat* or intervention* or counter* OR Pharmacokinetic* or Drug or drugs or Pharmacology or Medical or Nasal or Medication OR Scopolamine or Promethazine or Compazine or Phenergan or Ephedrine or Dextroamphetamine or Phenergan or Dexedrine or Meclizine or Cinnarizine or Dimenhydrinate OR Antidopaminergic* or "anti dopaminergic*" or Antihistaminergic* or "anti histaminergic*" or Anticholinergic* or "anti cholinergic*" or Placebo or Antivertig* or "anti vertig*")

#6 #2 OR #3

#7 #6 AND #4

#8 #7 OR #1

#9 #8 AND #5
